# Association Between Hospital Size and Outcomes of ES-SCLC Treated with Chemo-ICI: A Real-World Database Study in Japan

**DOI:** 10.3390/cancers18213555

**Published:** 2026-11-05

**Authors:** Yuta Yamanaka, Katsuhiko Iwasaki, Tatsuhiro Uenishi, Fumika Matsushita, Kinya Kokubo, Takayasu Kurata

**Affiliations:** 1 Department of Thoracic Oncology, Kansai Medical University, 2-5-1 Shinmachi, Hirakata 573-1010, Osaka, Japan; 2 Healthcare Consulting, Inc., Sumitomo Fudosan Chiyoda Fujimi Building, 1-8-19 Fujimi, Chiyoda-ku, Tokyo 102-0071, Japant-uenishi@hc-c.co.jp (T.U.);; 3 Faculty of International Political Science and Economics, Nishogakusha University, 6-16 Sanbancho, Chiyoda-ku, Tokyo 102-8336, Japan

**Keywords:** extensive-stage small-cell lung cancer, chemoimmunotherapy, immune checkpoint inhibitor, real-world data, hospital size, hematologic toxicity, time to treatment failure

## Abstract

**Background:** Chemotherapy combined with immune checkpoint inhibitors is the standard first-line treatment for extensive-stage small-cell lung cancer. We evaluated whether hospital size, as a surrogate for institutional healthcare resources, was associated with treatment outcomes. **Methods**: This retrospective observational study used a nationwide electronic medical records database in Japan. Patients with extensive-stage small-cell lung cancer (ES-SCLC) receiving first-line platinum–etoposide plus atezolizumab or durvalumab between 2019 and 2022 were included. Patients were classified by hospital size (<500 vs. ≥500 beds) and compared after propensity score matching. The primary endpoint was time to treatment failure (TTF), and secondary endpoints included grade ≥ 3 hematological toxicities and hepatic impairments. **Results:** After matching, each group included 81 patients. Median TTF was 4.8 months in the <500-bed group and 4.9 months in the ≥500-bed group. In crude analyses, severe leukopenia occurred in 67.9% of patients in smaller hospitals and 45.7% in larger hospitals (RR, 0.67; 95% CI, 0.51–0.89; *p =* 0.007), whereas severe neutropenia occurred in 79.0% and 58.0% of patients, respectively (RR, 0.73; 95% CI, 0.59–0.91; *p =* 0.006). In sensitivity analyses accounting for hospital-level clustering, the association for leukopenia was maintained, whereas that for neutropenia was no longer statistically significant. **Conclusions:** No clear difference in TTF was observed between the hospital-size groups. Exploratory safety analyses showed differences in severe hematological toxicities, although the findings were not fully consistent after accounting for hospital-level clustering. Further studies are needed to clarify the relationship between institutional healthcare resources and treatment outcomes.

## Simple Summary

Chemoimmunotherapy is the standard first-line treatment for extensive-stage small-cell lung cancer. However, little is known about whether treatment outcomes differ according to hospital size, which may serve as a surrogate for institutional healthcare resources. Using a nationwide electronic medical records database in Japan, we compared patients treated at smaller and larger hospitals. We found no clear difference in treatment effectiveness according to hospital size. Exploratory analyses showed differences in severe hematological toxicities between hospital-size groups, although these findings were not fully consistent after accounting for hospital-level clustering. These results suggest that some treatment outcomes may differ according to hospital size, but further studies are needed to clarify these relationships.

## 1. Introduction

Lung cancer remains the leading cause of cancer-related deaths worldwide, accounting for approximately 1.8 million deaths annually [1]. In Japan, lung cancer incidence has increased over recent decades and continues to be the leading cause of cancer mortality, with over 70,000 deaths reported in 2020 [2,3]. Among these, small-cell lung cancer (SCLC) constitutes 10–15% of all lung cancers in Japan, and approximately 70% of these cases are diagnosed as extensive-stage SCLC (ES-SCLC) [4]. In contrast to non-small-cell lung cancer (NSCLC), SCLC is commonly classified into two stages: limited stage (LS) and extensive stage (ES). LS is generally defined as disease confined to the hemithorax of origin, mediastinum, or supraclavicular nodes that can be encompassed within a tolerable radiation therapy field, whereas ES includes disease that has spread beyond these boundaries [5]. Among them, ES-SCLC is particularly aggressive and has a poor prognosis, with a 5-year relative survival rate of less than 10% reported by the National Cancer Institute [6]. Recent phase III trials have established chemoimmunotherapy, which combines chemotherapy with immune checkpoint inhibitors (chemo-ICI), as the standard first-line treatment for ES-SCLC [7,8]. However, treatment discontinuation or modification remains common in clinical practice, reflecting disease progression, adverse events, and patient-specific clinical considerations [9,10].

Several Japanese real-world studies have evaluated the effectiveness, safety, and treatment patterns of anti-programmed death-ligand 1 (PD-L1)-based chemo-ICI for ES-SCLC [9,10,11]. However, the outcomes were not stratified according to the hospital size or institutional resources. In England, a national audit-linked analysis showed that clinical trial participation rate, as a hospital-level factor, was associated with increased chemotherapy use among patients with SCLC [12]. In a US National Cancer Database analysis of stage IV NSCLC, patients treated at lower-volume hospitals (≤23 cases/year) had higher all-cause mortality than those at highest-volume hospitals (≥56 cases/year) after multivariable adjustment, indicating a volume–outcome association [13]. This broad relationship may be relevant to SCLC.

Therefore, we hypothesized that hospitals with well-established patient management systems and sufficient medical resources would have improved treatment outcomes for chemo-ICI, specifically reduced toxicities and improved treatment continuity. This study aimed to evaluate whether hospital size, as a surrogate marker for institutional healthcare resources, is associated with the treatment outcomes in patients with ES-SCLC receiving chemo-ICI.

## 2. Materials and Methods

### 2.1. Data Source

This retrospective observational study used real-world data (RWD) from the DATuM IDEA^®^ database (TOPPAN Holdings Inc., Tokyo, Japan), which is a nationwide resource that integrates electronic medical records from hospitals affiliated with the National Hospital Organization [14]. The database, constructed under the Next-Generation Medical Infrastructure Act to facilitate the secure secondary use of medical data, contains de-identified longitudinal information on approximately 980,000 individuals across 53 facilities [15]. The dataset was provided as anonymized (processed) medical information under Japan’s Next-Generation Medical Infrastructure Act after review and approval by the Processed Information Provision Review Committee of the Japan Medical Association Medical Information Management Organization (J-MIMO) [16] (initial approval/management number: 2024-2, approved on 20 May 2024; amended approval/management number: 2024-2-2, approved on 22 July 2025). The DATuM IDEA^®^ database is provided by TOPPAN Holdings Inc. and is not owned by the study sponsor, Healthcare Consulting, Inc.

### 2.2. Study Population

**Figure 1:**
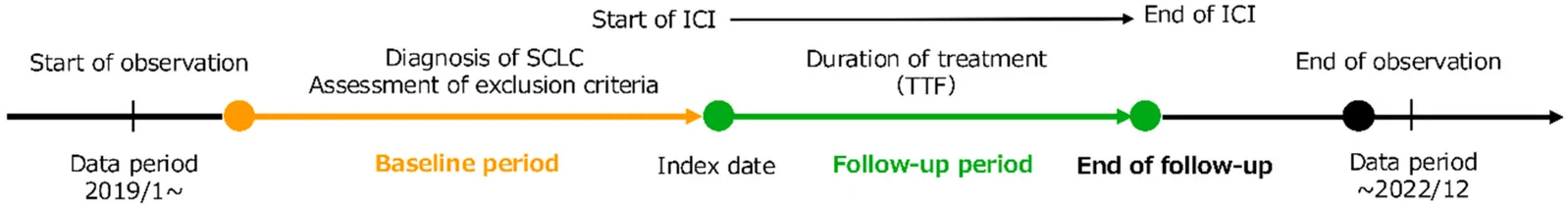
Study Timeline. Abbreviations: ICI, immune checkpoint inhibitor; SCLC, small-cell lung cancer; TTF, time to treatment failure.

Eligible patients were those diagnosed with ES-SCLC who received first-line treatment with a platinum–etoposide doublet (cisplatin or carboplatin plus etoposide) in combination with an immune checkpoint inhibitor (ICI) (atezolizumab or durvalumab) between 1 January 2019 and 31 December 2022. A visual overview of the study timeline is shown in Figure 1. The index date was defined as the first prescription of a chemo-ICI during the study period. The baseline period was defined as at least two weeks of available medical records prior to the index date, used to assess exclusion criteria, document comorbidities, and concomitant medications. Patients with prior prescriptions for epidermal growth factor receptor–tyrosine kinase inhibitors (EGFR-TKI), anaplastic lymphoma kinase–tyrosine kinase inhibitors (ALK-TKI), or other systemic chemotherapies during the baseline period were excluded. The follow-up period was extended from the index date until the earliest of the following: (i) initiation of second-line treatment with amrubicin or irinotecan, (ii) last administration date of ICI, or (iii) death. The end of the observation period was determined by either the study endpoint or the database cutoff, whichever occurred first.

### 2.3. Hospital Classification

Hospitals were classified into two categories according to bed capacity: those with fewer than 500 beds and those with 500 or more beds. In this study, hospital bed capacity was used as a practical indicator of hospital size and a surrogate for institutional healthcare resources. The threshold of 500 beds was selected with reference to the Japanese Ministry of Health, Labour and Welfare’s Patient Behavior Survey, which classified hospitals with 500 or more beds as a separate hospital category [17].

### 2.4. Propensity Score Matching

As structural differences between large and small hospitals may also be associated with variations in patient characteristics, we applied propensity score matching (PSM) to minimize confounding factors and enable valid comparisons between groups. Propensity scores were estimated using a logistic regression model that included ICI type, platinum agent (cisplatin or carboplatin), sex, age, Charlson Comorbidity Index (CCI) [18,19], baseline interstitial pneumonia, the use of proton pump inhibitors, antibiotics, and corticosteroids, and baseline duration. Patients were matched 1:1 using nearest-neighbor matching with a caliper of 0.2 and without replacement [20]. Propensity score matching was performed using the Matching package (version 4.10-15) in R (version 4.5.2; R Foundation for Statistical Computing, Vienna, Austria), with a caliper width of 0.2 standard deviations of the estimated propensity scores on the probability scale. Covariate balance before and after matching was assessed using standardized mean differences (SMDs) [21]. The CCI was calculated using the comorbidity package (version 1.1.0) in R [22], based on representative International Classification of Diseases, Tenth Revision (ICD-10) code ranges for each comorbidity category (see Supplementary Table S2). As the performance status was not available in the database we used, the CCI was used as a surrogate indicator of general health status.

### 2.5. Endpoints

The primary endpoint was the time to treatment failure (TTF), which was defined as the interval from the index date to the earliest amrubicin/irinotecan initiation, ICI discontinuation, or death. The reason for ICI discontinuation was not available in the database. Safety outcomes were assessed as exploratory secondary endpoints and included the incidence of grade ≥ 3 hematologic toxicities and hepatic impairments, as defined by Common Terminology Criteria for Adverse Events (CTCAE), version 5.0 [23] using sex-specific cut-off values (see Supplementary Table S1 for detailed definitions). Baseline interstitial pneumonia was defined as a recorded diagnosis within the three months preceding or on the index date. Interstitial pneumonia during follow-up was defined as a recorded diagnosis after the index date and on or before the end of follow-up, regardless of whether a diagnosis had been recorded before the index date. Thus, the follow-up outcome was not restricted to incident cases.

### 2.6. Statistical Analysis

For statistical analysis, survival outcomes were assessed using the Kaplan–Meier method, and group comparisons were conducted using the log-rank test. A Cox proportional hazards model was used to estimate the hazard ratio (HR) and 95% confidence interval (CI) for TTF between the hospital-size groups. Safety outcomes were compared between the groups using Fisher’s exact test, and crude risk ratios (RRs) with 95% CIs were calculated. Because the safety analyses were exploratory, no adjustment for multiple comparisons was performed.

To account for potential within-hospital clustering, sensitivity analyses were performed. For TTF, a shared frailty Cox model with hospital as a random effect was used [24]. A Cox model with hospital-level cluster-robust standard errors was also applied as an additional sensitivity analysis. For safety outcomes, modified Poisson regression with hospital-level cluster-robust standard errors was used to estimate cluster-adjusted RRs and 95% CIs [25,26]. Intraclass correlation coefficients (ICCs) for binary safety outcomes were estimated using the Fleiss–Cuzick estimator [27]. Statistical significance was set at *p* < 0.05.

## 3. Results

### 3.1. Patient Selection

**Figure 2:**
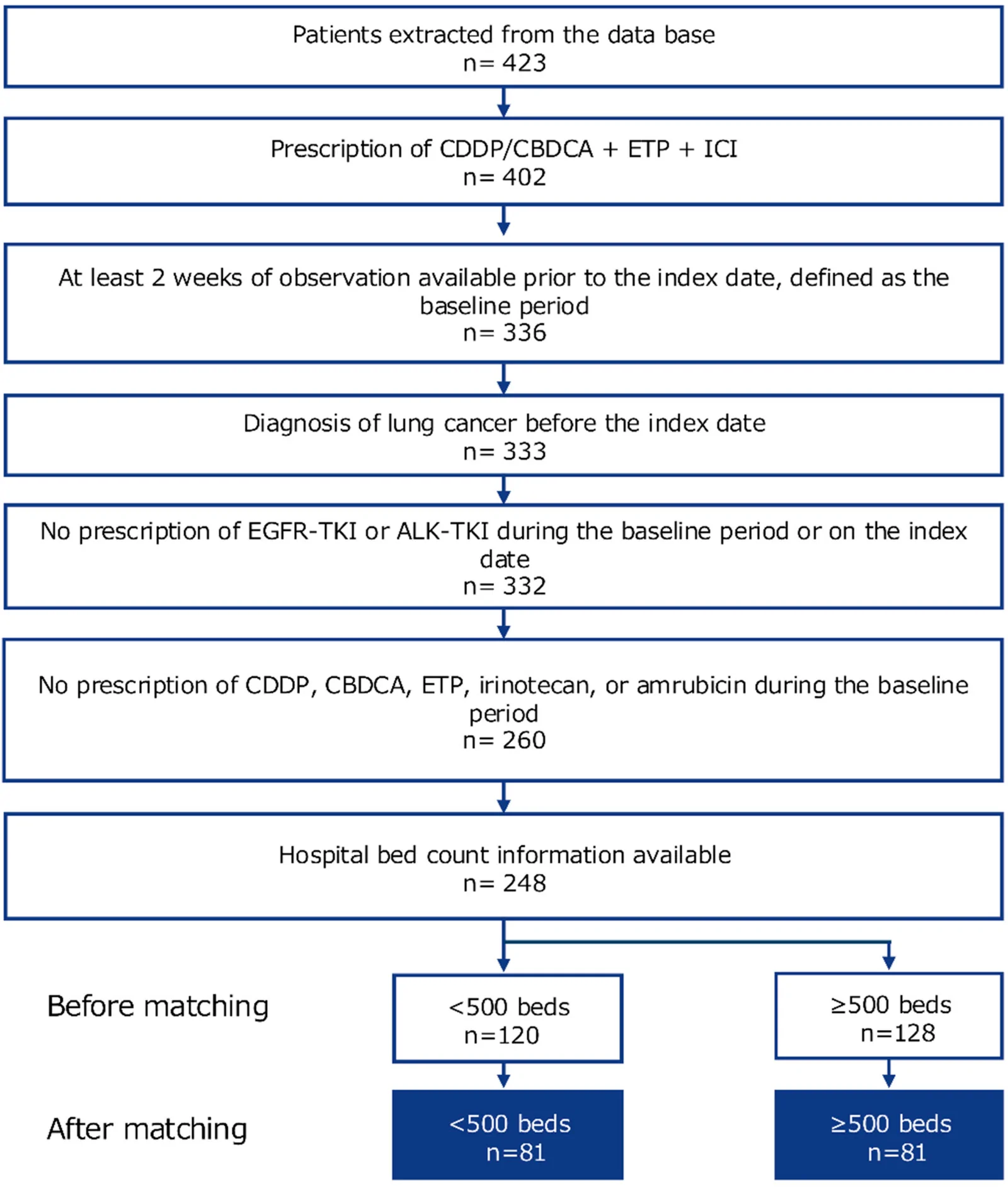
Flowchart of Patient Selection. Abbreviations: CDDP, cisplatin; CBDCA, carboplatin; ETP, etoposide; ICI, immune checkpoint inhibitor; EGFR-TKI, epidermal growth factor receptor–tyrosine kinase inhibitor; ALK-TKI, anaplastic lymphoma kinase–tyrosine kinase inhibitor.

We initially identified 423 patients with lung cancer who had received an immune checkpoint inhibitor (atezolizumab or durvalumab), a platinum agent (carboplatin or cisplatin), and etoposide, not necessarily administered on the same day. Of these, 402 had records indicating treatment with cisplatin or carboplatin plus etoposide in combination with ICI. Requiring at least two weeks of observation before the index date reduced the cohort to 336 patients. A total of 333 patients had a confirmed diagnosis of lung cancer before the index date. One patient who received EGFR-TKIs or ALK-TKI during the baseline period or on the index date was excluded, leaving 332 patients. An additional 72 patients prescribed other systemic chemotherapies (cisplatin, carboplatin, etoposide, irinotecan, or amrubicin) during the baseline period were excluded, resulting in 260 eligible patients. Of these, 248 patients had available hospital bed count information and were included in the final cohort. Before PSM, 120 patients were treated at hospitals with <500 beds and 128 at hospitals with ≥500 beds. After 1:1 PSM, 81 patients were included in each group for comparative analysis (Figure 2). In the propensity-score-matched cohort, the <500-bed group included patients from 17 hospitals and the ≥500-bed group included patients from 14 hospitals. The median number of patients per hospital was 5 (range, 1–11) and 6.5 (range, 1–13), respectively.

### 3.2. Baseline Characteristics

Baseline characteristics before propensity score matching are presented in Supplementary Table S3. After propensity score matching, the baseline characteristics were generally well balanced between groups (<500 beds group vs. ≥500 beds group): the mean age was 70.7 vs. 71.0 years, the proportion of males was 84% vs. 80%, and the mean CCI was 1.2 vs. 1.1. The treatment regimens were also comparable, with atezolizumab used in 65.4% vs. 61.7% and durvalumab in 34.6% vs. 38.3%. Carboplatin was the predominant platinum agent used in both groups. After PSM, the absolute SMD was <0.1 for 8 of the 10 covariates; the absolute SMDs for proton pump inhibitor and corticosteroid use were 0.102 and 0.128, respectively (Table 1).

### 3.3. Efficacy Outcomes

**Figure 3:**
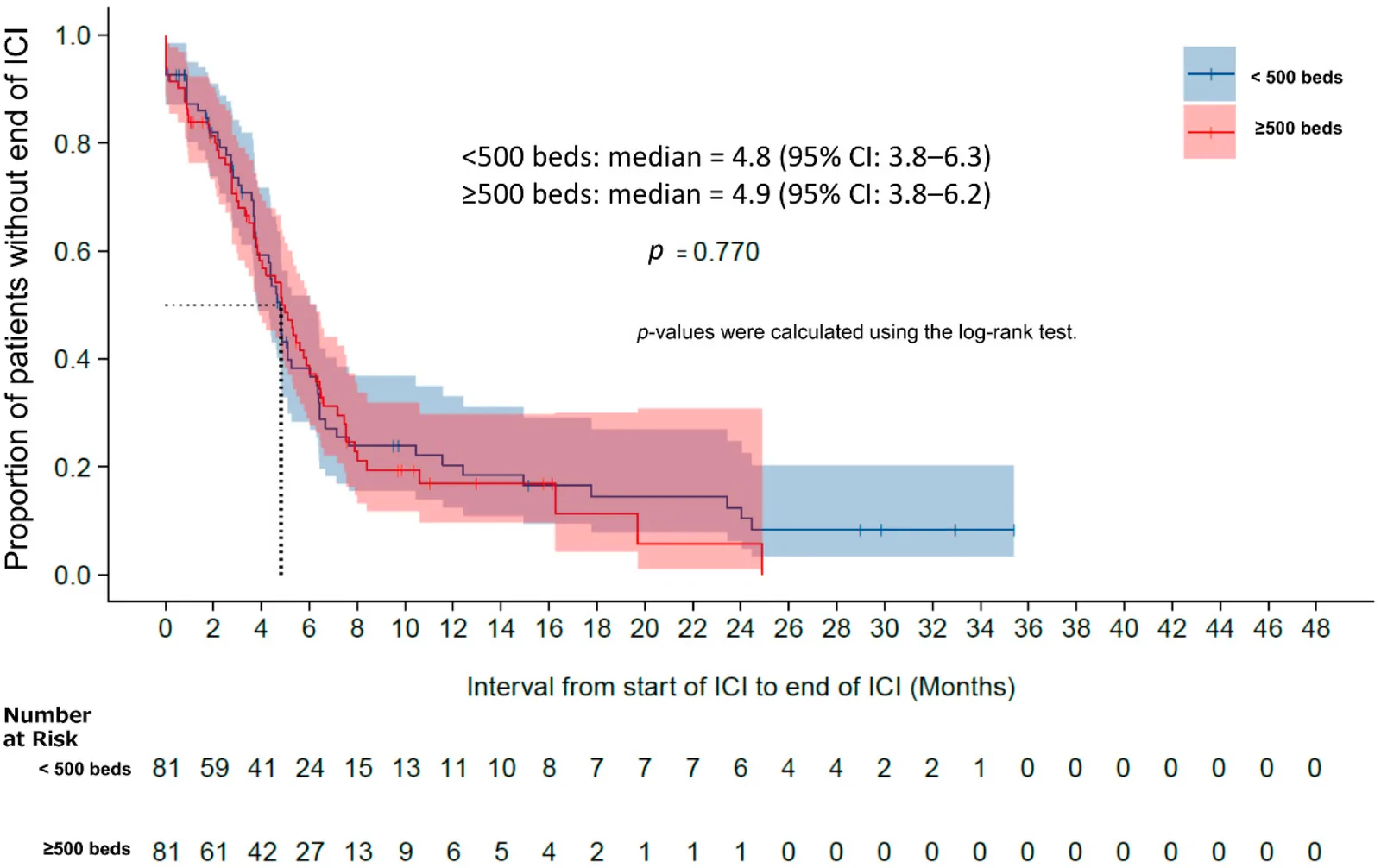
Kaplan–Meier Curves for TTF in the Two Groups. Abbreviations: CI, confidence interval; ICI, immune checkpoint inhibitor; TTF, time to treatment failure. The dashed lines indicate the 50% treatment continuation probability and the corresponding median TTF.

Kaplan–Meier analysis showed no significant difference in TTF between hospitals with <500 beds and those with ≥500 beds (median 4.8 vs. 4.9 months; 95% CI: 3.8–6.3 vs. 3.8–6.2; log-rank *p* = 0.77) (Figure 3). Consistent with the Kaplan–Meier analysis, the Cox proportional hazards analysis showed no significant difference in TTF between the two groups (HR, 1.05; 95% CI, 0.74–1.51; *p* = 0.773). The median follow-up duration was 4.32 months (interquartile range [IQR], 1.84–6.40) in the <500-bed group and 4.19 months (IQR, 2.07–6.52) in the ≥500-bed group. In sensitivity analyses accounting for hospital-level clustering, the results remained consistent. The shared frailty Cox model yielded an HR of 1.05 (95% CI, 0.74–1.51; *p* = 0.773), with a hospital-level frailty variance of 0.00008. Similarly, the Cox model with hospital-level cluster-robust standard errors yielded an HR of 1.05 (95% CI, 0.83–1.35; *p* = 0.671).

### 3.4. Safety Outcomes

Regarding safety, in the exploratory crude analysis, grade ≥ 3 leukopenia occurred in 67.9% of patients in the <500-bed group and 45.7% in the ≥500-bed group (RR, 0.67; 95% CI, 0.51–0.89; *p =* 0.007), while grade ≥ 3 neutropenia occurred in 79.0% and 58.0%, respectively (RR, 0.73; 95% CI, 0.59–0.91; *p =* 0.006). Based on ICD-10 diagnostic codes, interstitial pneumonia was observed in 0.0% of patients in the <500-bed group and 7.4% in the ≥500-bed group (*p =* 0.028); the RR was not estimable because no events occurred in the <500-bed group (Table 2).

In sensitivity analyses accounting for hospital-level clustering, the association for grade ≥ 3 white blood cell decrease was maintained (RR, 0.67; 95% CI, 0.50–0.90; *p =* 0.008), whereas the association for grade ≥ 3 neutropenia had a wider confidence interval and was no longer statistically significant (RR, 0.73; 95% CI, 0.50–1.08; *p =* 0.114). The estimated ICC for grade ≥ 3 leukopenia was approximately zero overall and within each hospital-size group (overall, −0.003; <500 beds, −0.066; ≥500 beds, −0.044). In contrast, positive within-hospital correlation was observed for grade ≥ 3 neutropenia (overall ICC, 0.280; <500 beds, 0.245; ≥500 beds, 0.244).

### 3.5. Exploratory Analysis

A post hoc exploratory analysis was conducted to examine potential factors associated with the observed differences in leukopenia and neutropenia between the hospital-size groups. No clear differences were observed in pegfilgrastim use during follow-up, hospitalization metrics (including the number of hospitalizations, total inpatient days, and average length of stay), or frequency of blood testing. The total monthly doses of carboplatin, cisplatin, and etoposide showed no clear differences between the groups, with slightly higher values observed in the ≥500-bed group. These exploratory findings did not identify clear differences in the assessed supportive care, monitoring, or chemotherapy-related measures that could account for the observed differences in hematological toxicities (Table 3).

## 4. Discussion

This study used DATuM IDEA^®^, a nationwide electronic medical record database in Japan, to compare the effectiveness and safety of first-line chemo-ICI for ES-SCLC in real-world clinical practice according to hospital size, used as a surrogate for institutional healthcare resources. Previous Japanese RWD studies have reported the effectiveness, safety, and treatment patterns of chemo-ICI containing anti-PD-L1 antibodies for ES-SCLC in real-world settings [9,10,11]. However, to our knowledge, no study has compared treatment outcomes from the perspective of hospital size as a surrogate for institutional healthcare resources, which represents the novelty of the present study.

In this study, outcomes were compared between the <500-bed and ≥500-bed groups after adjustment of patient characteristics using PSM. There was no significant difference in TTF between the two groups, and this finding remained consistent in sensitivity analyses accounting for hospital-level clustering. In contrast, the exploratory crude analyses showed lower frequencies of grade ≥ 3 leukopenia and neutropenia in the ≥500-bed group. In sensitivity analyses accounting for hospital-level clustering, the association for leukopenia was maintained, whereas that for neutropenia had a wider confidence interval and was no longer statistically significant. Although leukopenia and neutropenia are related outcomes, their estimated within-hospital correlations differed. This difference may partly explain the greater uncertainty in the cluster-adjusted estimate for neutropenia.

The differences observed according to hospital size in this study are generally consistent with previous reports suggesting that institutional factors may influence treatment delivery and outcomes in lung cancer. An analysis using national audit data from the United Kingdom showed that, in patients with SCLC, hospital-level factors as well as patient-level factors were associated with treatment delivery [12]. In addition, an analysis of stage IV NSCLC using the National Cancer Database in the United States reported that patients treated at high-volume facilities had a lower risk of death than those treated at low-volume facilities [13]. These findings support the rationale for the present study, namely that hospital size, as a surrogate for institutional healthcare resources, may be associated with differences in the delivery of lung cancer care.

In the present study, no clear difference in TTF was observed between the two groups. Because overall survival (OS) and progression-free survival (PFS) could not be assessed due to database-related constraints, TTF was used as an available endpoint. In real-world studies of NSCLC, time to treatment discontinuation (TTD) has increasingly been used as an effectiveness endpoint [28]. Lasala et al. [28] defined TTD as the time from treatment initiation to treatment discontinuation. In the present study, TTF was defined as the time to the earliest of initiation of second-line treatment, final administration of ICI, or death. Thus, the TTF used in this study is conceptually similar to TTD in that it is a real-world endpoint reflecting treatment continuation. However, TTF is affected by multiple factors, including disease progression, toxicity, treatment strategy, patient condition, and institutional practice patterns, and should therefore not be interpreted as equivalent to PFS or OS as an effectiveness endpoint [29,30].

Careful interpretation is required regarding the observed differences in hematologic toxicities between the two groups. To examine potential factors associated with the observed differences in hematologic toxicities, we conducted post hoc exploratory analyses. These analyses showed no clear differences in pegfilgrastim use during follow-up, hospitalization-related measures, frequency of blood tests, or monthly doses of carboplatin, cisplatin, and etoposide that could account for the observed differences in hematologic toxicities. Therefore, based on the present results alone, we cannot conclude that differences in supportive care, monitoring frequency, or anticancer drug dose accounted for the observed differences in hematologic toxicities. In addition, the observed differences may have been influenced not only by differences in the actual occurrence of toxicity but also by differences in opportunities for detection and recording of adverse events. In particular, the ≥500-bed group tended to have fewer inpatient days, which may have reduced opportunities to identify adverse events through tests and documentation during hospitalization. In addition, interstitial pneumonia was more frequent among patients treated at hospitals with ≥500 beds. This might reflect differences in opportunities for diagnosis and documentation, including access to respiratory specialists, whereas underdiagnosis or insufficient documentation may have occurred at smaller hospitals. This point suggests that incomplete capture of events dependent on clinical documentation may affect adverse event assessment in RWD studies [31,32].

This study has several limitations. First, the number of hospital beds was used as a measure of hospital size and as a surrogate for institutional healthcare resources. Therefore, this study did not directly evaluate institutional healthcare resources or clinical management, including staffing of specialized professionals, toxicity management protocols, or details of treatment decision-making processes. Second, given the information available in the database, sufficient variables for adjustment of SCLC patient characteristics could not be included as explanatory variables in the propensity score model, and potential differences in background characteristics between the two groups could not be fully adjusted. For example, performance status could not be obtained or adjusted for. Although CCI was used as a surrogate measure, CCI is an index of comorbidity burden and does not sufficiently reflect general condition, tumor burden, or treatment tolerability in patients with ES-SCLC [33]. In addition, factors reflecting disease status, such as liver, brain, and bone metastases, are considered useful for evaluating patient condition but could not be adjusted for in this study [34]. Data on PD-L1 status, weight loss, nutritional status, smoking history, and pleural or pericardial effusion were also unavailable. Baseline laboratory variables could not be consistently incorporated into the propensity score model because the timing of measurements relative to treatment initiation varied across patients. Detailed information on supportive care practices was also not available for consistent baseline adjustment. Therefore, residual confounding due to these unmeasured or incompletely measured factors may have influenced the observed associations. Third, information on initial dose adjustment was not available. Therefore, we cannot rule out the possibility that dose reduction or regimen modification at treatment initiation affected hematologic toxicities and TTF. Furthermore, dose intensity could not be rigorously evaluated because patient-specific standard doses and planned treatment intervals were not available in the database [35]. Fourth, because OS and PFS could not be assessed due to database-related constraints, TTF was used as the primary effectiveness endpoint in this study. OS is the most clinically important outcome in a disease with poor prognosis such as ES-SCLC, and PFS is also an endpoint that reflects tumor progression. Therefore, it should be noted that treatment effect cannot be fully evaluated using TTF alone. Although the analyses were independently programmed and cross-checked by two authors, both were affiliated with the study sponsor, and no sponsor-independent verification of the individual-level analyses was performed.

## 5. Conclusions

Based on Japanese RWD, this study showed no clear difference in TTF between the hospital-size groups, with hospital size used as a surrogate for institutional healthcare resources, whereas the observed frequency of grade ≥ 3 hematologic toxicities differed in the crude analyses. In sensitivity analyses accounting for hospital-level clustering, the association for leukopenia was maintained, whereas that for neutropenia had a wider confidence interval and was no longer statistically significant. However, exploratory analyses did not identify specific factors that explained these observed differences, and the influence of differences in opportunities for detection and recording cannot be ruled out. Therefore, the present findings do not demonstrate the superiority of treatment management at larger hospitals. Rather, they represent exploratory findings indicating the need to consider institutional healthcare resources and the limitations of adverse event capture in RWD when evaluating the safety of chemo-ICI for ES-SCLC. Further studies using more detailed data, including performance status, disease status, dose adjustment, supportive care, testing frequency, inpatient and outpatient management, as well as institutional healthcare resources and clinical management practices, are needed to evaluate how these factors relate to treatment outcomes.

## Figures and Tables

**Table 1:** Basic Characteristics of the Patients Receiving First-Line Chemoimmunotherapy According to Hospital Size.

	<500 Beds Group (*n* = 81)	≥500 Beds Group (*n* = 81)	SMD
**Immune Checkpoint Inhibitors (ICI)**			0.077
Atezolizumab (%)	65.4	61.7	
Durvalumab (%)	34.6	38.3	
**Platinum**			0.049
Carboplatin (%)	93.8	92.6	
Cisplatin (%)	6.2	7.4	
**Gender**			0.097
Male (%)	84.0	80.2	
Female (%)	16.0	19.8	
Age (mean (SD))	70.7 (8.5)	71.0 (6.9)	0.034
Charlson Comorbidity Index (mean (SD)) †	1.2 (1.1)	1.1 (1.0)	0.058
Baseline Duration (months), mean (SD)	14.5 (15.8)	14.2 (15.8)	0.017
Interstitial Pneumonia (%) †	3.7	4.9	0.061
Proton Pump Inhibitor Use (%) †	35.8	40.7	0.102
Antibiotic Use (%) †	46.9	48.1	0.025
Corticosteroid Use (%) †	7.4	11.1	0.128

Abbreviations: SD, standard deviation; SMD, standardized mean difference. † Assessed at baseline.

**Table 2:** Adverse Events (Derived from Laboratory Data) and ICD-10-based Diagnoses in the Two Groups.

	<500 Beds Group (*n* = 81)	≥500 Beds Group (*n* = 81)	RR (95% CI)	*p*-Value
**Evaluation based on lab data**				
AE (≥Grade 3): Hemoglobin decrease (anemia) (%)	12.3	13.6	1.10 (0.49–2.45)	1.000
AE (≥Grade 3): Leukopenia (%)	67.9	45.7	0.67 (0.51–0.89)	0.007
AE (≥Grade 3): Thrombocytopenia (%)	18.5	21.0	1.13 (0.61–2.11)	0.844
AE (≥Grade 3): Neutrophil count decreased (%)	79.0	58.0	0.73 (0.59–0.91)	0.006
AE (≥Grade 3): Aspartate aminotransferase increased (%)	1.2	2.5	2.00 (0.18–21.62)	1.000
AE (≥Grade 3): Alanine aminotransferase increased (%)	0.0	1.2	NE	1.000
**ICD-10-Based Diagnoses**				
Leukopenia (%)	1.2	1.2	1.00 (0.06–15.72)	1.000
Thrombocytopenia (%)	3.7	3.7	1.00 (0.21–4.81)	1.000
Neutropenia (%)	35.8	32.1	0.90 (0.58–1.38)	0.740
Interstitial Pneumonia (%)	0.0	7.4	NE	0.028

Abbreviations: AE, adverse event; AST, aspartate aminotransferase; ALT, alanine aminotransferase; CI, confidence interval; ICD-10, International Classification of Diseases, Tenth Revision; NE, not estimable; RR, risk ratio. Note: RRs were calculated for the ≥500-bed group relative to the <500-bed group. Table 2 presents unadjusted risk ratios. In sensitivity analyses accounting for hospital-level clustering, the risk ratios were 0.67 (95% CI, 0.50–0.90; *p* = 0.008) for grade ≥ 3 leukopenia and 0.73 (95% CI, 0.50–1.08; *p* = 0.114) for grade ≥ 3 neutropenia, as described in Section 3.4.

**Table 3:** Post hoc Exploratory Analysis of Hematologic Toxicities During Follow-up.

	<500 Beds Group (*n* = 81)	≥500 Beds Group (*n* = 81)	*p*-Value
Pegfilgrastim administration at least once during the follow-up period, (%)	45.7	38.3	0.426
Number of days of pegfilgrastim administration, median [IQR] ^a^	0.0 [3.0]	0.0 [2.0]	0.337
Number of hospitalizations per month, median [IQR] ^a^	0.8 [0.7]	0.7 [0.7]	0.626
Total number of inpatient days per month, median [IQR] ^a^	6.5 [6.0]	5.8 [6.5]	0.435
Average length of hospital stay, median [IQR] ^b^, ^§1^	10.0 [7.7]	9.2 [10.7]	0.527
Number of days with blood tests per month, median [IQR] ^a^	3.0 [2.1]	3.0 [2.4]	0.319
Total dose of carboplatin administered per month, median [IQR], mg ^c^, ^d^, ^§2^	345.1 [290.0]	360.0 [303.0]	0.323
Total dose of cisplatin administered per month, median [IQR], mg ^c^, ^d^, ^§3^	98.0 [24.3]	108.8 [69.1]	0.537
Total dose of etoposide administered per month, median [IQR], mg ^c^, ^d^	291.0 [274.1]	330.0 [278.8]	0.166

Abbreviations: IQR, interquartile range. Statistical analysis: Fisher’s exact test was used for pegfilgrastim administration, and the exact Wilcoxon rank sum test was used for all other variables. Footnotes: ^a^ The total number of days during the follow-up period was divided by the duration of the follow-up period to calculate monthly averages for each patient. ^b^ The average length of hospital stay was calculated by dividing the total number of inpatient days by the number of hospitalizations. ^c^ The total dose during the follow-up period was divided by the duration of the follow-up period to calculate monthly averages for each patient. ^d^ Dose reductions may have occurred not only due to adjustments for underweight patients but also due to treatment interruptions or planned dose reductions. ^§1^ Average length of hospital stay was calculated based on 74 patients in each group, as 7 patients were not hospitalized. ^§2^ Carboplatin use was calculated based on 76 patients in the <500 Beds Group and 75 patients in the ≥500 Beds Group. ^§3^ Cisplatin use was calculated based on five patients in the <500 Beds Group and 6 patients in the ≥500 Beds Group.

## Data Availability

Restrictions apply to the availability of these data. The data were obtained from the DATuM IDEA^®^ database and are not publicly available. Access to the data may be granted by the data provider upon reasonable request and with appropriate permission.

## Supplementary Materials

The following supporting information can be downloaded at https://www.mdpi.com/article/10.3390/cancers18213555/s1. Table S1: Definitions and threshold values used to identify Grade 3 hematologic toxicities and hepatic impairments according to CTCAE v5.0 criteria [23]. Table S2: Representative ICD-10 code ranges and corresponding weights for Charlson Comorbidity Index (CCI) conditions used in propensity score matching [36]. Table S3: Basic Characteristics of the Patients Receiving First-Line Chemoimmunotherapy According to Hospital Size Before Propensity Score Matching.

## Abbreviations

ALK-TKI	anaplastic lymphoma kinase–tyrosine kinase inhibitor
CCI	Charlson Comorbidity Index
CI	confidence interval
CTCAE	Common Terminology Criteria for Adverse Events
EGFR-TKI	epidermal growth factor receptor–tyrosine kinase inhibitor
ES	extensive stage
ES-SCLC	extensive-stage small-cell lung cancer
HR	hazard ratio
ICC	intraclass correlation coefficient
ICD-10	International Classification of Diseases, Tenth Revision
ICI	immune checkpoint inhibitor
IQR	interquartile range
LS	limited stage
NSCLC	non-small-cell lung cancer
OS	overall survival
PD-L1	programmed death-ligand 1
PFS	progression-free survival
PSM	propensity score matching
RR	risk ratio
RWD	real-world data
SCLC	small-cell lung cancer
SMD	standardized mean difference
TTD	time to treatment discontinuation
TTF	time to treatment failure
